# Adherence to 24-h movement guidelines in Spanish schoolchildren and its association with insulin resistance: a cross-sectional study

**DOI:** 10.3389/fpubh.2023.1146580

**Published:** 2023-07-10

**Authors:** María Dolores Salas-González, Laura M. Bermejo, Liliana G. González-Rodríguez, María Del Carmen Lozano-Estevan, Rosa M. Ortega, Ana M. López-Sobaler

**Affiliations:** ^1^Grupo de Investigación VALORNUT, Departamento de Nutrición y Ciencia de los Alimentos, Facultad de Farmacia, Universidad Complutense de Madrid, Madrid, Spain; ^2^Grupo de Investigación VALORNUT, Departamento de Nutrición y Ciencia de los Alimentos, Facultad de Farmacia, Universidad Complutense de Madrid, Instituto de Investigación Sanitaria del Hospital Clínico San Carlos (IdISSC), Madrid, Spain

**Keywords:** insulin resistance, children, schoolchildren, lifestyle behaviors, 24-h movement guidelines

## Abstract

**Introduction:**

Being more active, being less sedentary, and sleeping enough are associated with adequate body weight and adiposity in children. However, few researchers have analyzed these different lifestyle behaviors and the adherence to 24 h movement guidelines with respect to insulin resistance (IR) at school age. Therefore, we aimed to analyse the association between the adherence to 24 h movement guidelines (physical activity, sedentary time, sleep) and IR in Spanish schoolchildren.

**Methods:**

A cross-sectional study of 839 children (8–13 years, 51.1% girls) were studied. Anthropometric, biochemical, and lifestyle behavioral data were collected. IR was defined as HOMA-IR>3.16. Compliance with the 24 h movement guidelines were defined as ≥60 min/day of moderate and/or vigorous physical activity, < 120 min/day of screen time, and 9–11 h/day of sleep time. Associations between adherence to the 24 h movement guidelines and IR were performed by multivariate logistic regression analyses.

**Results:**

The IR in our sample was 5.0%, being higher in girls. Compliance with physical activity or screen time, but not with sleep recommendations, was associated with lower fasting glucose, fasting insulin, and HOMA-IR values. A low adherence to 24 h movement guidelines was associated with a higher risk of IR (odds ratio (95% CI): 2.150 [1.089–4.247]), especially in girls (odds ratio (95% CI): 2.800 [1.180–6.645]).

**Conclusion:**

Higher physical activity levels and lower screen times were associated with a lower risk of IR in schoolchildren, underlining the importance of adhering to as many healthy lifestyle recommendations as possible.

## 1. Introduction

Insulin resistance (IR) plays an important role in the pathogenesis of comorbid diseases associated with obesity, such as type 2 diabetes mellitus, coronary heart disease, and metabolic syndrome (1). IR has been reported as early as childhood and has been associated in children with alterations in other health parameters, such as dyslipidaemia or elevated blood pressure (2, 3).

There is a growing body of evidence to show the positive associations between different aspects related to the movement [sufficient physical activity (4), limited screen time (5), and appropriate sleep duration (6)] with the children and adolescent health.

Several studies have linked moderate and/or vigorous physical activity (MVPA) with a higher quality of life, better health status, and a lower prevalence of cardiometabolic diseases during school age and adolescence (4, 7–10).

Although an increased sedentary lifestyle has typically been associated with a lack of physical activity, the two can coexist dependently or independently (11). Regardless of physical activity, sedentary behavior has been associated with leading causes of mortality and risk of chronic diseases such as cardiovascular disease and type 2 diabetes mellitus (12, 13). Finally, sedentary behaviors such as TV watching, computer use and general screen time are common behaviors in childhood and adolescence, which are associated with increased body weight and alterations in lipid and glycemic profile (8).

Moreover, associations have been found between sleep deprivation and decreased insulin sensitivity, unhealthy eating habits, sedentary lifestyles, and overweight or obesity (6, 14, 15). Similarly, good sleep habits have also been shown to be a fundamental aspect of human health during all periods of life (16).

MVPA, decreased sedentary activities and adequate sleep duration and quality have been associated in adult reviews and meta-analyses with better weight status, lower RI, lower likelihood of type 2 diabetes mellitus and even lower mortality in several types of cardiovascular disease (17, 18). Furthermore, the relationship between these three aspects of lifestyle and body weight status and adiposity in children and adolescents is well-established (19–21).

Based on the evidence on child health, some organizations such as the World Health Organization have proposed recommendations on physical activity for children and adolescents (at least 60 min of moderate and/or vigorous physical activity) (22). The American Academy of Sleep Medicine has also proposed recommendations on adequate sleep for proper health (9–12 h per day for schoolchildren aged 6–12 years and 8–10 h per day for adolescents aged 13–18 years) (23). Moreover, Tremblay et al. (24) developed 24-h movement guidelines for children and youth, which integrated recommendations of three patterns: physical activity (at least 60 min of MVPA), sedentary time (< 120 min/day of screen time), and sleep duration (9–11 h/day) (24). Adherence to these guidelines has been associated with lower cardiometabolic risk (25) and overall health in children and adolescents (26, 27).

However, the relationship between different lifestyle patterns, separately and especially in combination, and IR at school-age has not been sufficiently studied, and the relationship remains unclear. Therefore, our aim with this study was to evaluate the lifestyle patterns of a group of Spanish schoolchildren and to analyze the association between the adherence to specific 24 h movement guidelines or a combination of them and IR at school age in order to contribute knowledge in this area and to evaluate the association between lifestyle and IR at school age.

## 2. Methods

### 2.1. Study sample

**Figure 1:**
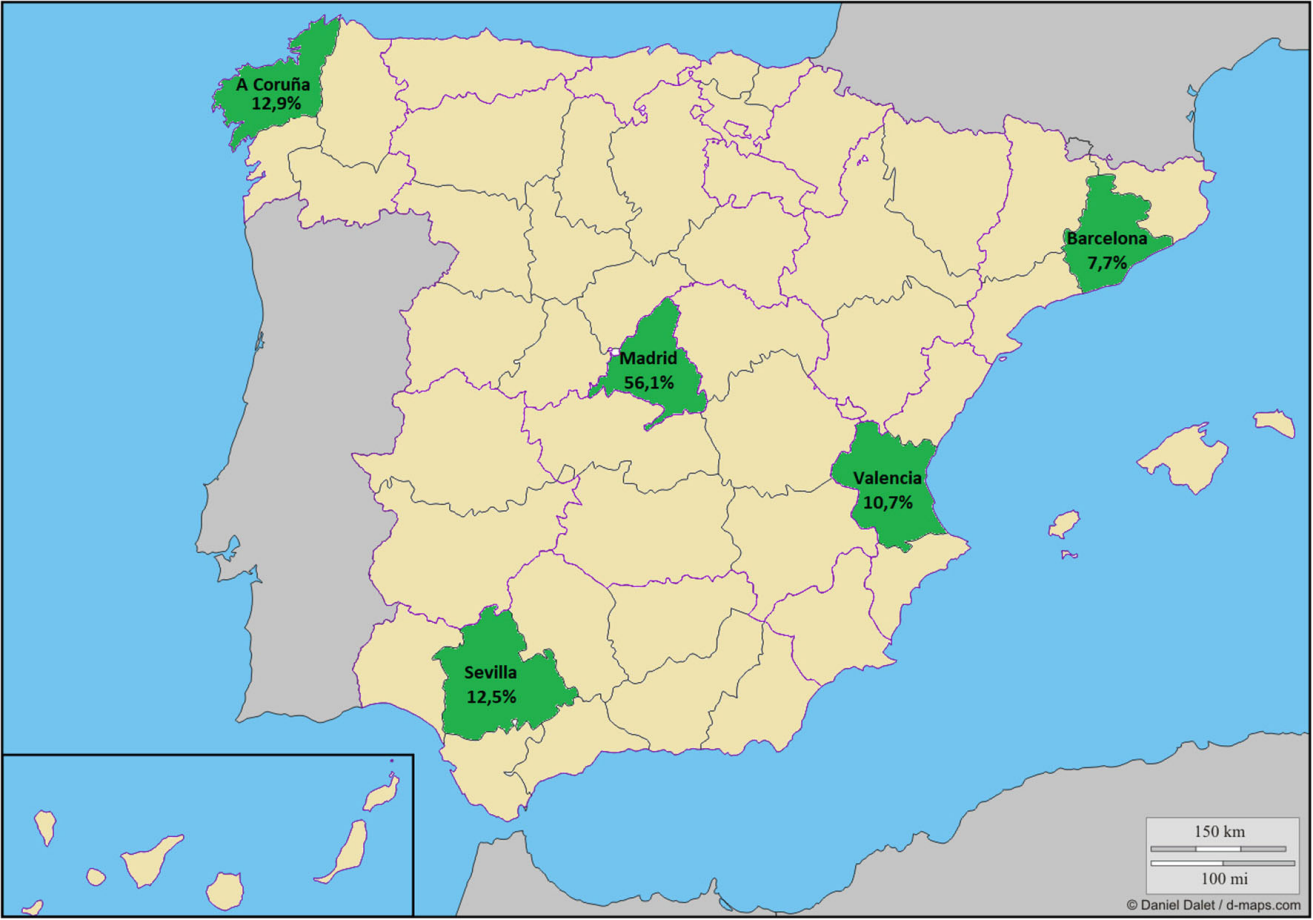
Sample distribution. Modified from “https://d-maps.com/carte.php?num_car=5672&lang=es”.

The cross-sectional observational study included a convenience sample of schoolchildren aged 8–13 years from five Spanish provinces (A Coruña, Barcelona, Madrid, Sevilla, and Valencia) (Figure 1). In each province, we randomly selected schools from a list of primary schools with at least two classes per grade. We contacted the management teams of 55 schools by telephone, and we arranged an interview to explain the characteristics and objectives of the study. Once we had obtained permission from the management teams (22 schools), we called the families of the children with ages in the target age range to a meeting to explain the aim and details of the study and to resolve any doubts they might have. Subsequently, we asked the parents or legal guardians of the schoolchildren to sign a written consent for their children to participate in the study.

The inclusion criteria were children aged between 8 and 13 years in the 4th, 5th, or 6th grade of primary school; written informed consent signed by the children's parents and/or guardians; and acceptance of all study conditions.

The exclusion criteria were presenting any disease that could modify the results of the study: metabolic or chronic diseases (diabetes, renal pathologies, etc.); impossibility of attending school on the days agreed for the tests; and receiving pharmacological treatment that could interfere with the results of the study. Measurements were made between February 2005 and June 2009.

**Figure 2:**
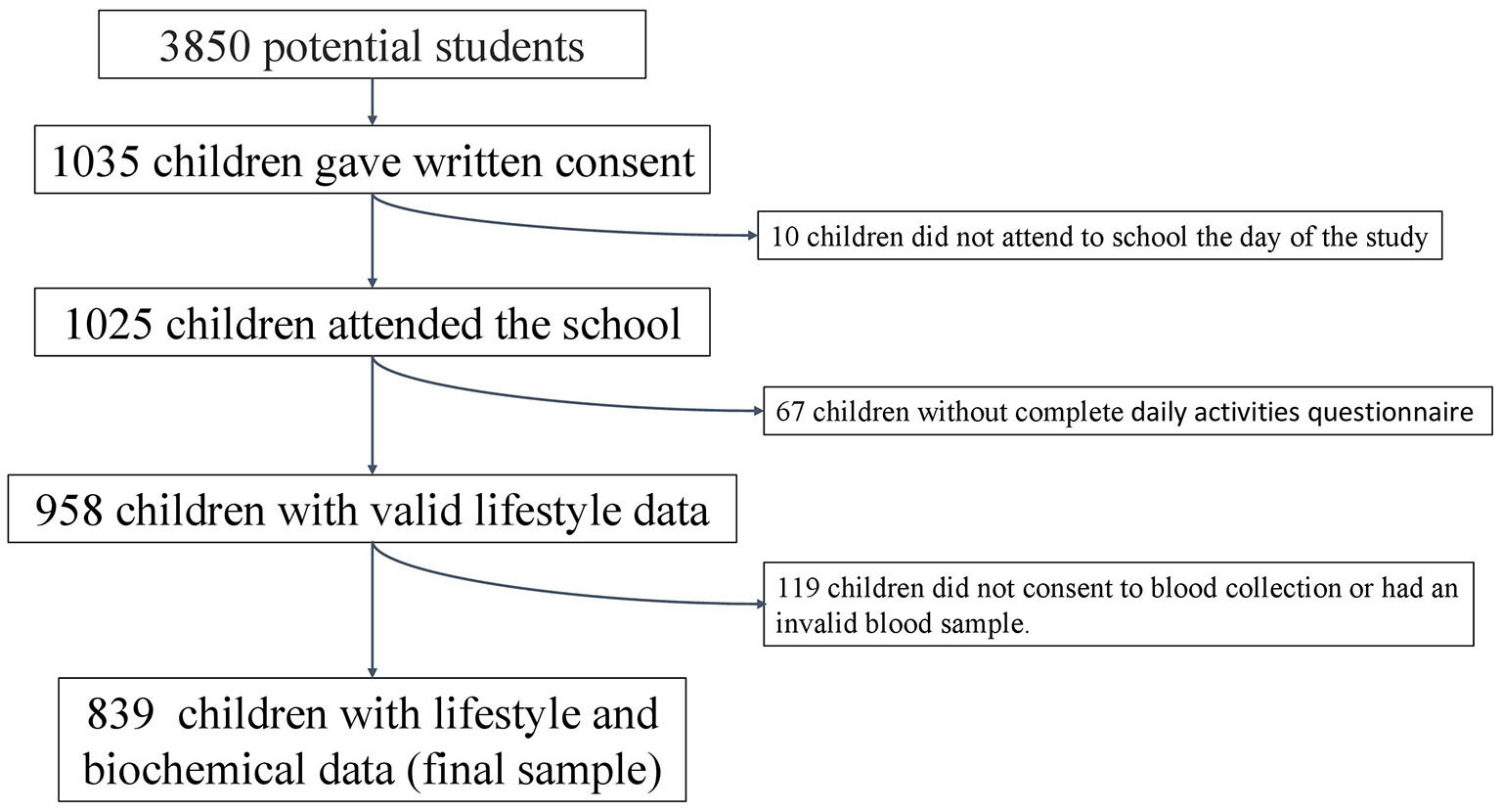
Flow chart of the selection process.

Our potential sample included 3,850 schoolchildren. This potential sample size was calculated based on the number of schools that had agreed to participate (22 schools), that each school could have from 1 to 3 different grade levels (4th, 5th, and/or 6th grade), that in some schools there is more than one classroom of the same level, and that the average number of students in each classroom is 25. Of these, 1,035 obtained written consent from their parents or guardians to participate in the study, so the approximate acceptance rate was 27%. Finally, 839 children had a complete daily activities questionnaire and valid biochemical data (Figure 2).

The participating children underwent a socio-demographic, lifestyle, anthropometric and biochemical study at the school by qualified research staff.

We conducted this study in accordance with the guidelines established in the Declaration of Helsinki, and all procedures involving human subjects/patients were approved by the Human Research Review Committee of the Faculty of Pharmacy of the Complutense University of Madrid (PI060318, approved on 17 March 2006).

### 2.2. Socio-demographic data

We used a self-completed survey by the parents/guardians of the children, including questions related to the demographic characteristics of the schoolchildren (sex, date of birth, and maximum level of education attained by the parents).

### 2.3. Anthropometric data

Anthropometric measurements (weight and height) were recorded by qualified personnel. The study was conducted in the schools during morning hours and following the standards established by the WHO (28). For the measurements, the subjects were barefoot and wore only underwear.

Weight and height were determined with a digital electronic scale (range: 0.1–150 kg, accuracy: 100 g; SECA ALPHA, GMBH, Igny, France) and a Harpenden digital stadiometer (range: 70–205 cm, accuracy: 1 mm; Pfifter, Carlstadt, NJ, USA). We calculated the body mass index (BMI) of the subjects using the formula weight (kg)/height^2^ (m^2^). Subsequently, we calculated the BMI z-score (zBMI) according to WHO growth standards (29).

### 2.4. Biochemical data

Blood samples were collected by venipuncture between 08:00 and 09:00 after 12 h of fasting. The nursing staff verified the adequacy of the fasting period before collecting the blood. Samples were collected at the school.

We colourimetrically determined plasma glucose levels by the glucose oxidase–peroxidase method (30) (Vitros GLU Slides, Rochester, NY, USA; CV = 2–8%). We measured insulin levels with an immunochemiluminometric assay (31) (Abbott Diagnostics, Madrid, Spain; CV = 4.8%). We used the homeostasis model assessment value (HOMA-IR) to reflect the degree of IR (32, 33): HOMA-IR = [basal glucose (mmol/L) × basal insulin (μU/mL)]/22.5. We considered IR present when the HOMA-IR was >3.16 (34).

### 2.5. The 24-h movement guidelines

We assessed lifestyle behaviors with an adapted daily activities questionnaire recording the children's usual daily activities, which had to be jointly completed by parents and children (35) and which has been used in previous studies (36–38). The questionnaire asked about the time spent on different activities usually carried out during the day: sleeping; being in class; studying; eating at different meals; playing sedentary games (PC, video game consoles) or watching TV; actively playing in the street and at home; in gymnastics or other sports activities at school; at recess and in extracurricular activities; and traveling between home and school and other activities, and the mode of transport used.

We then grouped the time spent on the different activities into the following categories: sleeping, very light activities (activities that are performed lying down, sitting, or standing, such as painting, playing an instrument, cooking, etc.); light activities (equivalent to walking on a flat surface at 4–5 km/h, such as cleaning the house, golf, table tennis, etc.); and moderate and/or vigorous activities (physical activities that require more energy expenditure such as cycling, skiing, tennis, dancing, basketball, football, rugby, running, etc.).

We classified children according to the 24 h movement guidelines (24) as “non-sedentary” if they restricted screen time to < 2 h/day (24) and as “physically active” if they performed at least 60 min of MVPA per day (24). We considered adequate sleep time if the child slept between 9 and 11 h (24).

The percentage of children complying with one, two, three or none of the recommendations of the 24 h movement guidelines was calculated. Schoolchildren were classified according to their adherence to a healthy lifestyle as follows: “low adherence”, if they complied with one or none of the recommendations of the 24 h movement guidelines, and “high adherence” if they complied with two or three recommendations.

### 2.6. Statistical analysis

Descriptive data are expressed as means and standard deviations for continuous variables and as percentages for categorical variables. We compared the data according to sex, lifestyle, and presence or absence of IR. We used the Kolmogorov–Smirnov test to determine the normality of the data. For comparison of means, we used the Mann–Whitney U-test if the distribution of variables was not normal and Student's t-test for normal distributions as well as the two-way ANOVA test. We compared categorical variables using the chi^2^ test and the Z-test of proportions. We calculated Spearman correlation coefficients between the hours spent on the different activities and HOMA-IR. We performed logistic regression analysis to identify risk or protective factors for IR, and we report odds ratios (OR) and 95% confidence intervals (CI). We considered a *p*-value of < 0.05 statistically significant. We performed all calculations with IBM SPSS Statistics for Windows, version 28.0 (Armonk, NY: IBM Corp, published in 2021).

### 2.7. *Post-hoc* power calculation

*Post-hoc* power analysis was calculated with G^*^Power (v. 3.1.9.6, Heinrich-Heine-Universität Düsseldorf, Düsseldorf, Germany) using an alpha error of 0.05, a sample size (n1= 367 and n2= 472) and an effect size of 0.35 to detect differences on HOMA-IR values according to adherence to the 24-h movement guidelines.

## 3. Results

These children were previously studied in another context (37, 38). The potential initial sample size was approximately 3,850 participants, which was calculated considering the number of schools that agreed to participate (*n* = 22), number of students per classroom (*n* = 25), and number of classrooms per grade (between two and three). Subsequently, 1,035 schoolchildren (49.2% boys) obtained written consent from their parents or guardians to participate in the study. Ten children were not in school on the day of the anthropometric study. We obtained valid lifestyle data from 958 schoolchildren (49.3% boys) and valid blood samples from 890 subjects (48.4% boys). The final sample was 839 individuals (48.9% boys) with complete biochemical and lifestyle data (Figure 2).

Table 1 shows the sociodemographic, anthropometric, biochemical, and lifestyle data of the total sample and by sex. We found no differences in age or parental education by sex. Boys had a higher zBMI and fasting glucose values than girls, but lower fasting insulin and HOMA-IR values. Of the children, 5% presented IR, the percentage being higher in girls (6.8 vs. 3.2%, *p* < 0.05). Boys spent more hours in front of the TV and computer, so they had more screen time than girls. Boys performed more MVPA than girls. 50.5% of schoolchildren spend < 2 h in front of the screen and only 31.9% of schoolchildren spend more than 1 h of MVPA per day. Overall, boys adhered less to screen time recommendations (45.9 vs. 55.0% for girls, *p* < 0.05) and more to physical activity recommendations (41.2 vs. 23.1%, *p* < 0.05) than girls. In addition, the recommendation on sleep is the one most frequently complied with by both sexes (82.4%), no differences between sex groups were seen.

**Table 1:** Anthropometric, biochemical, sociodemographic, and lifestyle parameters according to sex.

		**Total (*n =* 839)**	**Girls (*n =* 429)**	**Boys (*n =* 410)**
**Age and anthropometric data - X** ±**SD**				
Age (years)		10.1 ± 0.9	10.1 ± 0.9	10.1 ± 0.9
Weight (kg)		39.4 ± 9.3	39.5 ± 9.37	39.2 ± 9.3
Height (cm)		143.4 ± 0.1	144.0 ± 0.1	142.7 ± 0.1^*^
BMI (kg/m^2^)		19.0 ± 3.1	18.9 ± 3.0	19.1 ± 3.3
zBMI #		0.69 ± 1.13	0.56 ± 1.05	0.82 ± 1.20^*^
**Biochemical data - X** ±**SD**				
Glucose (mg/dL)		84.4 ± 9.7	83.4 ± 10.0	85.5 ± 9.2^*^
Insulin (mcU/mL)		6.2 ± 4.3	7.0 ± 4.8	5.5 ± 3.7^*^
HOMA-IR		1.31 ± 0.96	1.46 ± 1.07	1.16 ± 0.80^*^
Insulin Resistance [%(n)]		5.0 (42)	6.8 (29)	3.2 (13)^*^
**Parents' level of education [%(n)]**				
Father's highest level of education	No schooling or primary education	24.8 (208)	23.1 (99)	26.6 (109)
	Secondary education	34.7 (291)	35.2 (151)	34.1 (140)
	University studies	30.2 (253)	28.9 (124)	31.5 (129)
	Not determined	10.4 (87)	12.8 (55)	7.8 (32)^*^
Mother's highest level of education	No schooling or primary education	21.0 (176)	21.2 (91)	20.7 (85)
	Secondary education	39.7 (333)	39.2 (168)	40.2 (165)
	University studies	34.1 (286)	33.6 (144)	34.6 (142)
	Not determined	5.2 (44)	6.1 (26)	4.4 (18)
**Daily activities - X** ±**SD**				
Sleep (h/day)		9.31 ± 0.74	9.31 ± 0.75	9.32 ± 0.74
Very Light Physical Activity (h/day)		11.18 ± 1.64	11.17 ± 1.68	11.19 ± 1.61
Screen time (h/day)		1.81 ± 1.07	1.70 ± 1.06	1.92 ± 1.08^*^
TV (h/day)		1.42 ± 0.86	1.37 ± 0.87	1.48 ± 0.85^*^
PC (h/day)		0.33 ± 0.47	0.28 ± 0.44	0.39 ± 0.49^*^
Video games (h/day)		0.05 ± 0.20	0.04 ± 0.19	0.05 ± 0.21
Light Physical Activity (h/day)		2.64 ± 1.49	2.73 ± 1.55	2.53 ± 1.42
MVPA (h/day)		0.81 ± 0.48	0.73 ± 0.47	0.89 ± 0.48^*^
**Adherence to recommendations of 24h-movement guidelines [%(n)]**				
Screen time < 2 h/day		50.5 (424)	55.0 (236)	45.9 (188)^*^
MVPA ≥1 h/day		31.9 (268)	23.1 (99)	41.2 (169)^*^
Sleep between 9 and 11 h/day		82.4 (691)	82.5 (354)	82.2 (337)
Number of complied recommendations	0	9.1 (76)	9.3 (40)	8.8 (36)
	1	34.7 (291)	35.9 (154)	33.4 (137)
	2	38.6 (324)	39.6 (170)	37.6 (154)
	3	17.6 (148)	15.2 (65)	20.2 (83)
Adherence to 24-h movement guidelines	Low adherence	43.7 (367)	45.2 (194)	42.2 (173)
	High adherence	56.3 (472)	54.8 (235)	57.8 (237)

X, mean; SD, standard deviation; BMI, body mass index; MVPA, moderate and/or vigorous physical activity. Low adherence to 24 h movement guidelines: complied with one or none of recommendations; high adherence to 24 h movement guidelines: complied with two or three recommendations. Most variables followed nonparametric distribution; variables with normal distribution are marked (#). We used Mann–Whitney U-test for comparison of means if distribution of variables was not normal and Student's t-test for normal distribution. We analyzed associations between categorical variables with the χ^2^ test and the Z-test of proportions. Differences between sexes are indicated by asterisks (^*^p < 0.05).

Table 2 shows age, sociodemographic, anthropometric, and lifestyle data according to sex and presence of IR. We found no differences in age or parents' educational level according to IR status. Girls with IR spent more time on computers and screens in general and in very light activities, and they spent less time on light activities and MVPA than girls without IR. Therefore, fewer girls with IR met the 24 h movement guidelines for screen time (34.5 vs. 56.5%, *p* < 0.05) and physical activity (6.9 vs. 24.3%, *p* < 0.05) compared with girls without IR, with no difference in sleep time. In boys, we found no differences in their lifestyles based on having IR, except that boys with IR were less compliant with sleep recommendations than boys without IR (61.5 vs. 83.1%, *p* < 0.05).

**Table 2:** Anthropometric, sociodemographic, and lifestyle parameters according to sex and HOMA-IR.

		**Total**		**Girls**		**Boys**	
		**HOMA-IR** ≤ **3.16 (*****n** =* **797)**	**HOMA-IR** > **3.16 (*****n** =* **42)**	**HOMA-IR** ≤ **3.16 (*****n** =* **400)**	**HOMA-IR** > **3.16 (*****n** =* **29)**	**HOMA-IR** ≤ **3.16 (*****n** =* **397)**	**HOMA-IR**>**3.16 (*****n** =* **13)**
**Age and anthropometric data - X** ±**SD**							
Age (years)		10.1 ± 0.9	10.5 ± 1.0^*^	10.1 ± 0.9	10.4 ± 1.0	10.1 ± 0.9	10.5 ± 1.0
Weight (kg) – I		38.9 ± 9.0	47.7 ± 9.9^*^	39.0 ± 9.1	47.0 ± 9.1^*^	38.9 ± 9.0	49.2 ± 11.8^*^
Height (cm)# – I		143.1 ± 0.1	148.0 ± 0.1^*^	143.6 ± 0.1	149.2 ± 0.1^*^	142.6 ± 0.1	1454 ± 0.1
BMI (kg/m^2^) – IS		18.8 ± 3.0	21.7 ± 3.7^*^	18.7 ± 2.9	21.0 ± 2.9^*^	18.9 ± 3.1	23.2 ± 4.8^*^
zBMI – IS		0.64 ± 1.12	1.48 ± 1.12#^*^	0.51 ± 1.05	1.22 ± 0.83^*^	0.78 ± 1.17	2.04 ± 1.49#^*^
**Parents' level of education [%(n)]**							
Father's highest level of education	No schooling or primary education	24.7 (197)	26.2 (11)	22.8 (91)	27.6 (8)	26.7 (106)	23.1 (3)
	Secondary education	34.0 (271)	47.6 (20)	34.3 (137)	48.3 (14)	33.8 (134)	46.2 (6)
	University studies	30.7 (245)	19.0 (8)	29.5 (118)	20.7 (6)	32.0 (127)	15.4 (2)
	Not determined	10.5 (84)	7.1 (3)	13.5 (54)	3.4 (1)	7.6 (30)	15.4 (2)
Mother's highest level of education	No schooling or primary education	21.0 (167)	21.4 (9)	21.5 (86)	17.2 (5)	20.4 (81)	30.8 (4)
	Secondary education	39.0 (311)	52.4 (22)	38.0 (152)	55.2 (16)	40.1 (159)	46.2 (6)
	University studies	34.8 (277)	21.4 (9)	34.3 (137)	24.1 (7)	35.3 (140)	15.4 (2)
	Not determined	5.3 (42)	4.8 (2)	6.3 (25)	3.4 (1)	4.3 (17)	7.7 (1)
**Daily activities - X** ±**SD**							
Sleep (h/day)		9.32 ± 0.74	9.24 ± 0.79	9.31 ± 0.75	9.28 ± 0.76	9.32 ± 0.74	9.15 ± 0.88
Very Light Physical Activity (h/day) – I		11.13 ± 1.64	12.06 ± 1.49^*^	11.09 ± 1.68	12.25 ± 1.28#^*^	11.17 ± 1.60	11.64 ± 1.87#
Screen time (h/day)– I		1.78 ± 1.06	2.29 ± 1.18^*^	1.66 ± 1.04	2.26 ± 1.21^*^	1.90 ± 1.07	2.36 ± 1.18
TV (h/day)		1.41 ± 0.86	1.65 ± 0.85	1.35 ± 0.87	1.61 ± 0.90	1.47 ± 0.86	1.74 ± 0.74
PC (h/day)– I		0.32 ± 0.45	0.59 ± 0.70^*^	0.26 ± 0.41	0.59 ± 0.73^*^	0.38 ± 0.48	0.57 ± 0.64
Video games (h/day)		0.05 ± 0.20	0.05 ± 0.15	0.04 ± 0.19	0.05 ± 0.15	0.05 ± 0.21	0.05 ± 0.14
Light Physical Activity (h/day)– I		2.67 ± 1.49	1.99 ± 1.40^*^	2.79 ± 1.56	1.89 ± 1.17^*^	2.55 ± 1.41	2.21 ± 1.85
MVPA (h/day) – S		0.81 ± 0.48	0.69 ± 0.48^*^	0.74 ± 0.47	0.57 ± 0.42^*^	0.89 ± 0.48	0.95 ± 0.50
**Adherence to recommendations of 24-h movement guidelines [%(n)]**							
Screen time < 2 h/day		51.3 (409)	35.7 (15)^*^	56.5 (226)	34.5 (10)^*^	46.1 (183)	38.5 (5)
MVPA ≥1 h/day		32.7 (261)	16.7 (7)^*^	24.3 (97)	6.9 (2)^*^	41.3 (164)	38.5 (5)
Sleep between 9 and 11 h/day		82.9 (661)	73.8 (31)	82.8 (331)	79.3 (23)	83.1 (330)	61.5 (8)^*^
Number of complied recommendations	0	8.9 (71)	11.9 (5)	9.3 (37)	10.3 (3)	8.6 (34)	15.4 (2)
	1	33.6 (268)	54.8 (23)^*^	34.0 (136)	62.1 (18)^*^	33.2 (132)	38.5 (5)
	2	39.1 (312)	28.6 (12)	40.8 (163)	24.1 (7)	37.5 (149)	38.5 (5)
	3	18.3 (146)	4.8 (2)^*^	16.0 (64)	3.4 (1)	20.7 (82)	7.7 (1)
Adherence to 24-h movement guidelines	Low adherence	42.5 (339)	66.7 (28)^*^	43.3 (173)	72.4 (21)^*^	41.8 (166)	53.8 (7)
	High adherence	57.5 (458)	33.3 (14)^*^	56.8 (227)	27.6 (8)^*^	58.2 (231)	46.2 (6)

X, mean; SD, standard deviation; BMI, body mass index; MVPA, moderate and/or vigorous physical activity. Low adherence to 24 h movement guidelines: complied with one or none of recommendations; high adherence to 24 h movement guidelines: complied with two or three recommendations. Most variables followed nonparametric distribution; variables with normal distribution are marked (#). We used Mann–Whitney U-test for comparison of means if the distribution of variables was not homogeneous, and Student's t-test for homogeneous distributions. We analyzed associations between categorical variables with χ^2^ test and Z-test of proportions. Significant differences are indicated by asterisks (^*^p < 0.05). Two-way ANOVA analysis: S: differences by sex; I: differences by insulin resistance (IR) score; R: interaction between sex and IR.

Because only two children with IR complied with all three lifestyle recommendations, we decided to group subjects into low adherence (adherence to one or none of the recommendations) and high adherence (adherence to two or three recommendations) for statistical analysis. A lower percentage of girls with IR had high adherence to the 24 h movement guidelines compared with girls without IR (27.6 vs. 56.8%, *p* < 0.05). We observed no significant differences based on the presence of IR in the boys.

Table 3 shows the different biochemical parameters and IR according to adherence to the 24 h movement guidelines. Children who did not follow the recommendations for screen time or physical activity and those with lower adherence to the 24 h movement guidelines had higher fasting insulin levels and HOMA-IR values. We found no significant differences according to the sleep recommendations for either sex.

**Table 3:** Biochemical parameters according to sex and lifestyle parameters.

	**Total**		**Girls**		**Boys**	
**Screen time**						
	≥ 2h/day (*n =* 415)	< 2 h/day (*n =* 424)	≥ 2 h/day (*n =* 193)	< 2 h/day (*n =* 236)	≥ 2 h/day (*n =* 222)	< 2 h/day (*n =* 188)
Glucose (mg/dL) – S	84.8 ± 9.7	83.5 ± 9.4^*^	83.3 ± 10.7	83.0 ± 9.1	86.1 ± 8.4	84.1 ± 9.6^*^
Insulin (mcU/mL) – SA	6.9 ± 4.8	5.8 ± 4.8^*^	7.9 ± 5.7	6.6 ± 5.5^*^	6.1 ± 3.7	4.8 ± 3.5^*^
HOMA-IR – SA	1.46 ± 1.07	1.17 ± 0.81^*^	1.65 ± 1.29	1.30 ± 0.82^*^	1.29 ± 0.80	1.00 ± 0.76^*^
**MVPA**						
	< 1 h/day (*n =* 571)	≥ 1 h/day (*n =* 268)	< 1 h/day (*n =* 330)	≥ 1 h/day (*n =* 99)	< 1 h/day (*n =* 241)	≥ 1 h/day (*n =* 169)
Glucose (mg/dL) – SA	84.7 ± 8.9	83.0 ± 10.8^*^	83.9 ± 9.7	80.6 ± 10.0^*^	85.8 ± 7.4	84.4 ± 11.0
Insulin (mcU/mL) – SA	6.7 ± 4.5	5.5 ± 5.3^*^	7.4 ± 5.0	6.4 ± 7.3^*^	5.8 ± 3.6	5.0 ± 3.6^*^
HOMA-IR – SA	1.42 ± 1.01	1.09 ± 0.79^*^	1.55 ± 1.13	1.15 ± 0.79^*^	1.24 ± 0.80	1.05 ± 0.79^*^
**Sleep time**						
	< 9 h/day or >11 h/day (*n =* 148)	9–11 h/day (*n =* 691)	< 9 h/day or >11 h/day (*n =* 75)	9–11 h/day (*n =* 354)	< 9 h/day or >11 h/day (*n =* 73)	9–11 h/day (*n =* 338)
Glucose (mg/dL) – S	85.3 ± 10.1	84.3 ± 9.6	84.1 ± 11.6	83.2 ± 9.7	86.5 ± 8.2	85.3 ± 9.4
Insulin (mcU/mL) – S	6.4 ± 4.5	6.2 ± 4.3	6.9 ± 4.6	7.0 ± 4.8	5.9 ± 4.3	5.4 ± 3.5
HOMA-IR – S	1.37 ± 0.99	1.30 ± 0.95	1.47 ± 1.04	1.45 ± 1.08	1.26 ± 0.92	1.14 ± 0.77
**Adherence to 24-h movement guidelines**						
	Low adherence (*n =* 367)	High adherence (*n =* 472)	Low adherence (*n =* 194)	High adherence (*n =* 235)	Low adherence (*n =* 173)	High adherence (*n =* 237)
Glucose (mg/dL) – SA	85.1 ± 9.3	83.4 ± 9.6^*^	84.0 ± 10.7	82.4 ± 9.1	86.3 ± 7.4	84.4 ± 10.1^*^
Insulin (mcU/mL) – SA	7.1 ± 5.0	5.7 ± 4.7^*^	7.9 ± 5.7	6.5 ± 5.5^*^	6.2 ± 3.8	5.0 ± 3.5^*^
HOMA-IR – SA	1.50 ± 1.12	1.16 ± 0.78^*^	1.67 ± 1.30	1.28 ± 0.80^*^	1.32 ± 0.83	1.04 ± 0.76^*^

Data are shown as mean ± standard deviation; MVPA: moderate and/or vigorous physical activity. Low adherence to 24 h movement guidelines: complied with one or none of recommendations. High adherence to 24 h movement guidelines: complied with two or three recommendations. Two-way ANOVA analysis: S: differences according to sex; A: differences according to adherence to lifestyle recommendations; R: interaction between sex and adherence to lifestyle recommendations. No variable followed a normal distribution. For within-sex differences between IR and non-IR, we used Mann–Whitney U-test. Significant differences are shown with asterisks (^*^p < 0.05).

HOMA-IR directly and significantly correlated with screen time and very light physical activity, and indirectly correlated with light physical activity, MVPA, sleep time, and the number of recommendations met. These associations were maintained when boys and girls were separately analyzed, except for the sleep time (Table 4).

**Table 4:** Correlations between lifestyle variables and HOMA-IR.

	**Total (839)**	**Girls (429)**	**Boys (410)**
Sleep (h/day)	−0.074^*^	–0.086	–0.067
Very Light Physical Activity (h/day)	0.256^*^	0.273^*^	0.251^*^
Screen time (h/day)	0.213^*^	0.215^*^	0.267^*^
TV (h/day)	0.147^*^	0.138^*^	0.194^*^
PC (h/day)	0.192^*^	0.239^*^	0.199^*^
Video games (h/day)	0.137^*^	0.098^*^	0.180^*^
Light Physical Activity (h/day)	−0.207^*^	−0.217^*^	−0.231^*^
MVPA (h/day)	−0.158^*^	−0.127^*^	−0.136^*^
Number of recommendations complied of the 24-h movement guidelines	−0.182^*^	−0.174^*^	−0.185^*^

Data shown are Spearman's rho. MVPA: moderate and/or vigorous physical activity. Significance is shown with asterisks (^*^p < 0.05).

The results of logistic regression analysis showed that low adherence to 24 h movement guidelines was associated with increased risk of IR both in the total sample and in girls. In girls, non-adherence to screen time or physical activity recommendations was associated with an increased risk of IR in the crude model, but the association disappeared when corrected for age and zBMI. There was no association with sleep time. In addition, no significant association was found in boys (Table 5).

**Table 5:** Associations between lifestyle parameters and IR by sex. Logistic regression analysis.

	**Total (839)**		**Girls (429)**		**Boys (410)**	
	**Model 1 OR 95% CI**	**Model 2OR95% CI**	**Model 1 OR 95% CI**	**Model 2OR95% IC**	**Model 1 OR 95% CI**	**Model 2OR95% CI**
**Sreen time**						
< 2 h/day	1	1	1	1	1	1
≥ 2 h/day	1.897 (0.994–3.621)	1.499(0.763–2.946)	2.468 (1.119–5.442)^*^	1.850(0.810–4.224)	1.368 (0.440–4.255)	1.128(0.345–3.690)
**MVPA**						
≥ 1 h/day	1	1	1	1	1	1
< 1 h/day	2.435 (1.067–5.555)^*^	2.029(0.845–4.874)	4.322 (1.009–18.506)^*^	3.914(0.884–17.317)	1.126 (0.362–3.504)	1.236(0.371–4.121)
**Sleep time**						
9–11 h/day	1	1	1	1	1	1
< 9 h/day or >11 h/day	1.725 (0.846–3.516)	1.423(0.672–3.014)	1.251 (0.491–3.188)	0.978(0.370–2.588)	3.078 (0.977–9.701)	3.487(0.996–12.214)
**Adherence to 24-h movement guidelines**						
High adherence	1	1	1	1	1	1
Low adherence	2.702 (1.401–5.211)^*^	2.150(1.089–4.247)^*^	3.444 (1.490–7.962)^*^	2.800(1.180–6.645)^*^	1.623 (0.536–4.919)	1.529(0.481–4.861)

Model 1. Crude model. Model 2. Adjusted for age, sex (in total sample) and zBMI. MVPA: moderate and/or vigorous physical activity. Low adherence to 24 h movement guidelines: complied with one or none of recommendations. High adherence to 24 h movement guidelines: complied with two or three recommendations. Significance is shown with asterisks (^*^p < 0.05).

## 4. Discussion

Our findings showed that schoolchildren with IR had a less active lifestyle in general and had higher levels of screen time than those without IR, especially girls. Children who met the recommendations for physical activity or screen time or had higher adherence to 24 h movement guidelines had lower fasting glucose, fasting insulin, and HOMA-IR levels. Finally, increased adherence to lifestyle recommendations was associated with a lower risk of IR.

In our study, 43.7% of schoolchildren had low adherence to 24 h movement guidelines (complying with only one or none of the recommendations), similar to that reported in another Spanish child population (39.2%) (39) and lower than that reported in an international study (62.9%) (40). The low adherence to lifestyle recommendations in our study was mainly due to the low adherence to physical activity recommendations, because it was lower than the adherence to sleep and screen time recommendations.

About half of our schoolchildren adhered to the screen time guidelines, spending < 2 h in front of a screen. This figure is difficult to compare with those in other studies as the percentage of children meeting screen time recommendations widely varies from 8.1 to 98.4% in the 6–12 years age group, as shown by a recent meta-analysis (41). However, approximately half of the sample dedicated more hours to sedentary leisure than is recommended.

The time devoted by our population to MVPA was 49 min, being higher in boys. These data are similar to those published in a study on Spanish children aged 7 to 11 years (44 min) (39) and are lower than those reported in a Finnish child population aged 3–6 years (1 h 25 min) (42). Only 31.9% of the sample spent at least 1 h on MVPA, and this figure was even lower for girls.

In our study and in the bibliography, schoolchildren spend too much time on sedentary leisure (41) and less time than recommended on physical activity (39, 40). This indicates that, in general, schoolchildren are sedentary and inactive, and policies should be strengthened to reverse this situation, which can lead to serious health problems. Moreover, boys and girls have different lifestyles, with boys being more active but more sedentary. Therefore, recommendations and interventions should be tailored to sex to ensure greater adherence.

The recommendation of the 24 h movement guidelines most followed by our schoolchildren was for sleep time, as observed in other studies (36, 42). The children in our study slept an average of 9 h 19 min, lower than that reported in Spanish children in the ALADINO-2019 study (10 h 17 min) (43), but similar to that of a U.S. study of children aged 9–10 years (44) and in a Swedish study of children 10 to 12 years old (9 h) (45). Sleep recommendations were not met by 17.5% of children, a figure higher than that of other European countries (46). This is surprising because, according to the COSI strategy, Spanish children are among those who spend more time sleeping compared with other European countries (47), although this difference may be due to the COSI strategy being applied in children aged 6–9 years, an age somewhat younger than ours, and children sleep less as they age (48).

Regarding the relationship of compliance with the 24 h movement guidelines to IR, sedentary schoolchildren in our study (≥2 h/day of screen time) had higher HOMA-IR values. This result is consistent with those of the European IDEFICS study of children aged 2–17 years (49) and in English (7–13 years) (50) and Spanish schoolchildren (5–14 years) (51). This potential relationship between sedentary lifestyles and IR may have been due to less muscle activity and energy expenditure, causing alterations in homeostasis. Spending more time in sedentary activities may result in a lower rate of glucose processing in skeletal muscle, accompanied by less inhibition of glucose production in the liver (52).

In addition, our schoolchildren who performed more than 60 min of MVPA per day had lower HOMA-IR values. In a study of Indian children, physical activity during early adolescence was found to play a protective role against IR (53). The IDEFICS study assessed cross-sectional associations between physical activity and the clustering of cardiometabolic risk factors (such as IR), and moderate activity time negatively correlated with cardiometabolic risk score (54). Physical activity can influence IR through various potential mechanisms, one of which is the absence of skeletal muscle contractions being associated with reduced blood flow, which decreases circulating glucose transport to muscle (55). MVPA is associated with increased glucose uptake by skeletal muscle, leading to a decrease in blood glucose (56).

Considering sleep as part of a healthy lifestyle to prevent IR is important, as a lack of sleep is associated with elevated stress levels as well as hypothalamic–pituitary–adrenal regulation, which may lead to neuroendocrine disruption, which in turn may cause dysregulation in glucose–insulin metabolism (57). However, the results of studies on the relationship between sleep and IR in children and adolescents are controversial. In our study, sleep duration was not associated with IR, as observed in the IDEFICS study, conducted on children aged 2–15 years (58), and in the HELENA study, conducted on adolescents aged 12.5–17.5 years (59). However, this differs from the results of the ABCD Growth Study, conducted in Brazil on adolescents aged 11–18 years, where this component was the only one related to HOMA-IR (60). A narrative review in a pediatric population concluded that the relationship between sleep and HOMA-IR is not yet clearly established, although “convincing evidence” exists of an association between sleep duration and biomarkers of type 2 diabetes mellitus (including HOMA-IR) (61). The lack of association in our study could be explained by the high percentage of children who adhered to the sleep recommendation, which was the most followed guideline.

Similar to our results, Werneck et al. found that having several inappropriate lifestyle behaviors, or low adherence to recommendations, increases the risk of elevated HOMA-IR, although in this study, in addition to the lifestyle behaviors we analyzed, skipping breakfast was also considered (60).

Therefore, our study agrees with other studies in the relationship between screen time and physical activity with lower IR (49–51, 53, 54). While on the relationship between sleep duration and IR, the results remain controversial, with some studies finding a relationship (60, 61), and others, such as ours and other studies (58, 59), finding no association.

However, to our knowledge, only Werneck et al. (60) and ourselves have jointly assessed these parameters in relation to IR and we have both observed that adherence to various lifestyle factors is more robustly related to IR. Therefore, this is one of the first studies to assess a possible closer relationship by jointly assessing these lifestyle parameters in association with IR.

As other authors have pointed out, our results showed that examining the relationship between a single lifestyle behavior and health is not appropriate. Therefore, different lifestyle recommendations should be combined to further examine the relationship between lifestyle and health outcomes, such as cardiometabolic risk or IR (25–27).

Since this is one of the first studies to jointly assess lifestyle in relation to IR in children. And the results suggest that analyzing lifestyle factors separately may not address the problem of IR, a pooled analysis should be conducted to analyze the risk of IR. In addition, physical activity and sedentary time seem to be more related to IR than sleep in this study sample.

Given that IR is related not only to obesity but also to other comorbidities since childhood and based on the results of our work. It is important to carry out health policies, recommendations and studies that are not limited to including increased physical activity or decreased sedentary lifestyle, but should be comprehensive, including more physical activity, less sedentary leisure (such as screens nowadays) and correct sleep habits, as these activities should be approached as a whole.

One of the main strengths of our study is the large sample size, although the sample is a convenience one and therefore not representative of the Spanish population.

One of the main limitations was that being a large study, we could not perform Tanner's clinical staging and did not obtain information on the pubertal status of the participants, which could have influenced the prevalence of IR. Moreover, as we used a questionnaire to measure lifestyle and no other methods, such as accelerometers, the data may have been overestimated. Another possible limitation was that, as only a small sample followed all the recommendations, they had to be arbitrarily grouped for the analysis. Ethnicity was not collected in the study, and approximately half of the subjects did not answer about their country of origin, although the majority of those who reported were Spanish. Finally, our study had a cross-sectional design that highlighted the existence of associations but cannot demonstrate causal relationships and the sample studied is a convenience one, so the conclusions of our study should be confirmed in future research. Furthermore, although the sample size was not calculated a priori, a *post hoc* power analysis was performed and showed that our study had a high power (99%) to detect differences in mean HOMA-IR values according to adherence to 24 h movement patterns. Nevertheless, it would have been desirable to determine the sample size a priori and to use a probability sampling method.

Future lines of research are based on carrying out an intervention study in Spanish schoolchildren of the same age to verify the results obtained. In this study we intend to use an accelerometer in addition to the lifestyle questionnaire, in order to validate the questionnaire and to have non-self-reported lifestyle data.

In conclusion, we found that about half of the schoolchildren had low adherence to the 24 h movement guidelines. Sleep time was the most followed recommendation, and physical activity was the least. Girls were less physically active but also spent less time on screens. Our study showed that higher adherence to recommended lifestyle patterns was inversely associated with IR, especially in girls and when several lifestyle factors were combined. Intervention studies are needed that can confirm the possible protective effect of increasing adherence to the 24 h movement guidelines on IR in schoolchildren.

## Data availability statement

The datasets analysed for this study are available upon reasonable request from MDS-G (masala06@ucm.es).

## Ethics statement

The present study protocol was reviewed and approved by the Ethics Committee of the Faculty of Pharmacy of the Complutense University of Madrid, in Spain (PI060318, approved on 17 March 2006). Written informed consent to participate in this study was provided by the participants' legal guardian/next of kin.

## Author contributions

AML-S and RMO contributed to conception and design of the study. MDS-G, LMB, MCL-E, and LGG-R collected and processed data. MDS-G performed the statistical analysis and wrote the first draft of the manuscript. All authors contributed to the article and approved the submitted version.
